# Efforts to link HIV-positive and high-risk blood donors to HIV testing, and treatment services, Mozambique, 2019–2020

**DOI:** 10.1038/s41598-025-03259-2

**Published:** 2025-07-01

**Authors:** Udhayashankar Kanagasabai, Leonardo Sousa, Michelle S. Chevalier, Steve Gutreuter, Dina Ibraimo, Sara Salimo, Eva Naueia, Laison Daniel, Selma Khan, Dawud Ujamma, Stephanie Behel, Inacio Malimane, Bakary Drammeh

**Affiliations:** 1 Divsion of Global HIV and TB, Centers for Disease Control and Prevention, Corporate Square, MS E-04, Atlanta, GA 30329 USA; 2 Division of Global HIV and TB, Centers for Disease Control and Prevention, Maputo, Mozambique; 3 U.S. State Department, Washington, DC USA; 4 Servico Nacional de Sangue, Maputo, Mozambique; 5 Ariel Foundation, Maputo, Mozambique; 6 Centro de Colaboração em Saúde, Maputo, Mozambique; 7 Present Address: Center for Scientific Review, National Institute of Health, Maryland, USA

**Keywords:** Blood donors, Linkage, HIV, Transfusions, Diseases, Health care, Medical research, Risk factors, Retrovirus

## Abstract

Mozambique’s National Blood Transfusion Services (NBTS) is tasked with providing safe and available blood but also conducting systematic screening of at-risk potential donors, notifying seropositive blood donors, and linking them to HIV care and treatment services. Potential blood donors who were deferred from donating following a behavioral risk screening and all blood donors who screened seropositive for HIV were notified and offered linkage to HIV testing, care, and treatment services by community-based organizations. A prospective study among HIV-positive blood donors and deferred donors was conducted from May 2019 to July 2020 at Maputo Central Hospital Blood Bank and the National Reference Blood Center. The associations between testing, initiating care and treatment services among HIV-positive blood donors and prospective deferred donors were estimated using fully Bayesian multivariable logistic models and odds ratios. Among 885 prospective blood donors enrolled, 173 (20%) were deferred due to self-reported high-risk behaviors identified through a screening questionnaire, and 712 (80%) passed the behavioral-risk screening tool, donated, and the blood donation tested positive for HIV. There were more than 2.5 times as many male donors as female donors with a positive HIV test, and among the deferred donors, more than 84% were males. 36% (256/712) of seropositive donors and 35% (61/173) of deferred donors were referred to HIV testing services. 62% (158/256) of seropositive donors and 4.9% (3/61) of deferred donors who were successfully referred were linked to care and treatment services, and 96% (152/158) of these seropositive donors and 100% (3/3) of deferred as high-risk donors initiated antiretroviral therapy (ART). Of the three service organizations used, one outperformed the other two in linking seropositive donors to ART treatment. The NBTS can serve as a critical entry point for identifying HIV-positive persons. Improved implementation of risk behavior screening tools is needed and could contribute to early identification and initiation of ART for potential donors. Innovative strategies and solutions by community-based organizations can be used to improve blood donor notification and linkage to HIV testing and treatment services.

## Introduction

The World Health Organization (WHO) has recommended various strategies to improve global blood safety and availability^1,2^, but Sub-Saharan Africa (SSA) has struggled to achieve the WHO recommended target of 10 whole blood donations per 1000 population^1,3–5^. SSA countries face several challenges, including (1) recruitment and retention of voluntary non-remunerated donors; (2) hemovigilance systems; and (3) timely notification and linkage of donors to HIV testing (HTS) and HIV care and treatment services (HCT)^6,7^.

Every blood donation carries a risk of transmitting blood-borne pathogens, such as HIV, Hepatitis B Virus, Hepatitis C Virus, and syphilis^2^. In 2019, SSA had 25.6 million people living with HIV, with 59% of new HIV infections globally^2,9^. Globally, the WHO estimates that up to 3% of new HIV infections are due to contaminated blood products^8^. The U.S. President’s Emergency Plan for AIDS Relief (PEPFAR) has supported 14 African and Caribbean countries with high HIV prevalence in strengthening their national blood transfusion services^6,8^. Screening potential blood donors for HIV and reducing transfusion-transmitted infections (TTIs) [HIV, syphilis, Hepatitis B, and Hepatitis C) has remained a public health priority in most PEPFAR-supported countries.

Although medical screening and testing for HIV among blood donors has improved in many resource-constrained countries, there is no standardized system for notifying blood donors of their HIV results and linking them to HIV Testing and HIV Care and Treatment services^9–12^. Linkage to care is a crucial early step in successful HIV treatment and is typically defined as the completion of a first medical visit after an HIV diagnosis^13,14^. Without proper follow-up, these individuals may not be aware of their HIV status and miss out on opportunities for early diagnosis and treatment, which can have significant implications for their health and the prevention of further transmission. One study has estimated that globally, prompt identification of HIV-infected individuals and initiation of anti-retroviral therapy (ART) may avert up to 21 million AIDS-related deaths and 28 million new infections by 2030^15^. Due to limited resources, many national blood services cannot adequately follow up with HIV seropositive blood donors for linkage to HTS and HCT services^10,13,14,16,17^. The existing donor notification system requires donors to contact the blood bank to request the results of their HIV test and places the onus on the donors’ desires to know their infection status or disclose their HIV status. A study conducted in 2009 among the general population at a provincial hospital in Mozambique found that 9.5% of potential blood donors were deferred from donating when using a behavioral risk screening questionnaire. The same study also found a 10.6% seroprevalence for HIV and 8.5% seroprevalence for all other TTIs among these potential blood donors^18^. National HIV policy requires donors who test positive for HIV at blood banks to be linked to HTS, where confirmatory testing is done with further linkage to HCT. Currently, Mozambique lacks a standardized approach for the referral of deferred “high risk” blood donors to HTS, and notification of donors testing HIV positive and linkage to HTS and HCT. To date, there have only been three studies conducted in west Africa and one in east Africa that describes linkage of potential donors to HTS and HCT services^10,16,19,20^.

This study aimed to improve linkage mechanisms for deferred “high risk” individuals and HIV-reactive blood donors to HIV testing and care and treatment services and explore the feasibility of using community-based organizations to improve notification of HIV status and linkage to HTS and HCT services of blood donors.

## Methods

### Study design and setting

A prospective blood donor study was conducted at Maputo Central Hospital Blood Bank and the National Reference Blood Center (SENASA) from May 2019 to July 2020. The SENASA conducts blood collections at its permanent fixed facility and mobile blood collection sites in and around Maputo City. All clients ages 13 years and above seeking to donate blood at the study sites during the study period were screened, and those who met the criteria were invited to participate in the study. All study participants provided informed written consent as per SENESA requirements and those below the age of 18 provided assent with consent from their legal guardians. This study followed the Reporting of Observational Studies in Epidemiology (STROBE) guidelines. All research was performed per relevant guidelines/regulations.

This activity was reviewed by the U.S. Centers for Disease Control and Prevention, deemed not research, and was conducted consistent with applicable federal law and CDC policy (45 C.F.R. part 46.102(l)(2), 21 C.F.R. part 56; 42 U.S.C. Section 241(d); 5 U.S.C. Sect. 552a; 44 U.S.C. Section 3501 et seq). The protocol was approved by the Mozambique Ethics Review Board.

### Study population

As part of routine practice, all potential blood donors undergo systematic pre-donation behavioral screening based on WHO guidance conducted by a trained nurse or blood bank staff^6,8^. This includes recording demographic information, previous donation history, and risk factors for TTIs (e.g., sex with a commercial sex worker, injection drug use, recent sexually transmitted infections) to determine possible deferral from blood donation. Per Mozambique guidelines, all potential blood donors who presented at a blood center for blood donation provided consent to have their HIV-positive results returned to them for the purpose of linkage to care. All clients seeking to donate blood at the study sites during the study period were screened, and those who met the study criteria were enrolled in the study.

*Inclusion criteria*

- All consenting blood donors who donated blood at SENASA and Maputo Central Hospital Blood Bank and their mobile collection sites and tested positive for HIV.
- All consenting potential blood donors deferred through the behavioral screening questionnaire because they were “at risk” for TTI (e.g., having engaged in commercial sex work, injecting drugs, had a recent STI in the past 12 months are deferred for six months).

*Exclusion criteria*

- All blood donors who donated blood at the SENASA and Maputo Central Hospital Blood Bank and tested negative for HIV.
- All individuals who refused consent to be contacted by the organizations involved in the study were excluded from the study.

### Intervention

**Fig. 1:**
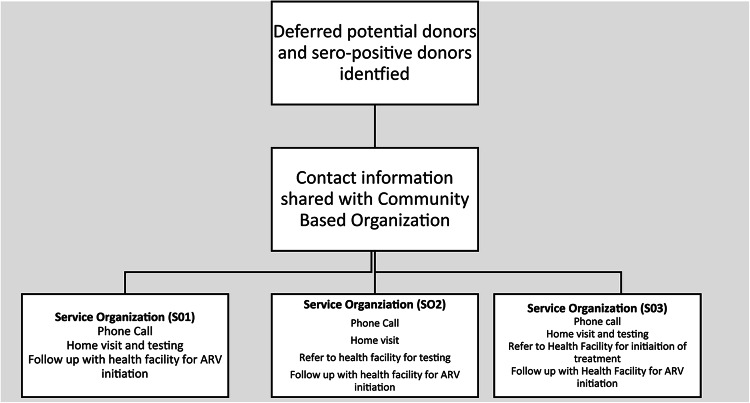
Study Linkage framework for linkage of HIV positive and at high-risk blood donors

To establish notification of testing results, referral, and initiation of services for deferred “high-risk” donors and HIV positive donors to HTS and HCT services, in collaboration with SENASA, three community-based organizations (CBO) SO1, SO2 and SO3 were engaged as partners to facilitate linkage (Fig. 1.). All three community-based organizations were well established and respected entities that had a proven record of working in Mozambique for several years, especially in HIV programming. Deferred individuals who were considered at high-risk for TTIs during the pre-donation screening process and HIV-positive blood donors were identified by staff at the study sites for further follow-up by the CBO field staff. No personal identifying information (names or addresses) beyond client phone numbers was shared on a weekly basis with the community-based partners for follow-up.

For follow-up, the three CBOs engaged healthcare providers and community health workers in Maputo City and relevant provinces to follow up with study participants to ensure that these individuals receive necessary testing and/or treatment. The follow-up process of study participants involved making up to three phone calls in a week at different times using the number provided during the pre-donation screening process. Study participants who answered their phones were read a scripted message encouraging them to get tested at the nearest HIV testing site. No participants with HIV positive results were informed via phone calls. Initial contact rates with participants did not vary by organizations. Two service organizations (SO1 and SO3) performed home HIV testing for consenting participants. Community health care workers met participants who consented to testing at the agreed-upon HIV testing location (including home-based testing) and guided them through the process of testing and ART initiation if they tested positive. To maintain confidentiality, no personal identifying information (names or addresses) beyond client phone numbers was shared with CBOs. All study participants were linked using unique study identifiers and phone numbers. Lost to follow-up for this study was defined “as the client not being able to be contacted in linked to the appropriate services”.

### Data collection

Blood bank staff were hired and trained to extract data daily from the ledgers. Data abstractors collected data using paper data collection tools. Initial data collected from blood bank records included demographic data, phone number, screening questionnaire responses, and TTI test results. Follow-up data completed by the community-based organizations collected the following information: contact made with the Follow-up data completed by the community-based organizations collected the following information: contact made with the participant, participant tested, test result or referred to testing services, participant-initiated ART. Both forms were entered into the database and linked via their unique study IDs. Double data entry was performed to enter data into a Microsoft access database daily. All forms were kept in a secure locked cabinet only accessible by the study coordinator.

### Data analysis

The associations between those who initiated testing and treatment among HIV-positive persons who donated blood and prospective donors were estimated using Bayesian multivariable logistic models. The outcome variable was a binary indicator for initiation on ART (0 = no; 1 = yes). Our covariates of interest were the service organizations (identified only as SO1–SO3), age group (13–19, 20–29, 30–39 and 40–64, inclusive), sex, and donor type (donors who contributed HIV-positive blood and high-risk donors who were deferred pending HIV testing), and the donor’s choice of donation venue (permanent facility or mobile). We first assessed all covariates using bivariate logistic regression for significance at 95%. We then generated a full multivariable logistic model, including a full model of all covariates with uncertainty intervals for odds ratios that excluded 1.0 and their two-way interactions. Subsequent model simplification was based on leave-one-out cross-validation (LOO)^21^, which is an improvement over the deviance information criterion (DIC). Like DIC or AIC (Akaike information criterion), smaller values of LOO indicate better predicting models. The final model for inference had the smallest LOO and retained only essential covariates.

All computations were implemented using R 4.0.3^22^, and the logistic regression models were implemented using the Rstanarm package^23^, which uses Hamiltonian Monte Carlo to produce samples from the joint posterior distribution of the model parameters given the data and prior distributions. We based inference on four Monte Carlo chains of 10,000 draws each after 10,000 warm-up draws from the joint posterior distribution. We used Student-t distributions with four degrees of freedom as weakly informative priors on the logistic coefficients. Those distributions are robust to any extreme values that might occur. Uncertainty intervals for the parameter estimates were obtained as the 2.5th and 97.5th percentiles of the posterior samples which provide 0.95 probability intervals for the true but unknown values of the parameters given the data and priors. The probabilities of linkage to care were obtained from the posterior distribution from the final multivariable model.

## Results

**Fig. 2:**
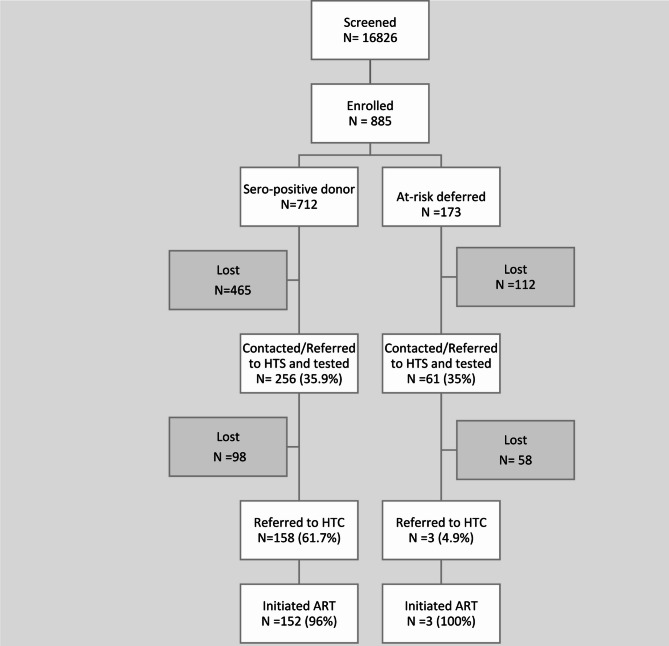
Recruitment identification of potential blood donors that are lab-confirmed HIV positive and or screened at ‘at-risk’ blood donors and linkage to HIV services flowchart

Among the 885 prospective blood donors enrolled, 712 (80%) contributed donations which were found to be seropositive, and the blood was discarded (Fig. 2). Among these, 256/712 (35.9%) were linked to HTS for diagnosis and 158 of these 256 (61.7%) were linked to HIV care and treatment. Of these, 152 (96%) initiated ART. There were 173 (20%) prospective donors identified as having elevated risk for HIV infection through the screening questionnaire prior to donation and were deferred pending HIV testing. Among the 173 deferred, 61(35%) were linked to HTS, and /three (4.9%) tested HIV positive; all were linked to care and initiated on ART.

In total, there were 2.52 times more male donors (634) than female donors (251) (Table 1). Among those testing HIV positive at the blood center, there were more than 2.5 times as many male donors as female donors, and among the deferred donors, 84% were males (Table 1). 72% of the donors were between 20 and 39 years old. Among the 792 HIV positive donors and prospective donors who presented at permanent facilities, 21.3% were deemed as high risk for TTIs from the behavioral survey and were deferred. In contrast, only 4.3% of those potential donors who presented at mobile sites were deemed high risk for TTIs from the surveys.

**Table 1:** Demographic characteristics of prospective blood donors in Maputo City and Maputo Province, Mozambique

Demographic group		Positive	Deferred	Total
Sex	Females	224	27	251
	Males	488	146	634
	Both	712	173	885
Age	(13,19)	31	9	40
	(20,29)	231	59	290
	(30,39)	291	60	351
	(40,65)	159	45	204
	All ages	712	173	885
Venue choice	Permanent facility	623	169	792
	Mobile	89	4	93
	Both	712	173	885

There was no evidence of association between age group and donation venue and linkage to HIV treatment in bivariate logistic models (Table 2), and those covariates were not considered further. The two-way interactions and the main effect of sex were not significant in multivariable logistic models and were sequentially eliminated. The final model included only the main effects of blood-donation service organization and donor type. The adjusted odds ratios in the final model (Table 2) show that SO2 and SO3 were not as successful as SO1 in linking HIV-positive donors to ART treatment (Fig. 2). The true odds ratio for linkage by SO2 relative to SO1 is between 0.3 and 0.5 with 0.95 probability. The true odds ratio for ART initiation by SO3 relative to SO1 is between 0.1 and 0.3 with 0.95 probability. The odds of linkage to care among high-risk prospective donors who were deferred from donation are lower than the odds of linkage to care among donors who donated HIV-positive blood. Specifically, the odds ratio is 0.2, which indicates that the odds of linkage to care among deferred high-risk prospective donors are 0.2 times the odds of linkage to care among HIV-positive blood donors. Additionally, the probability range of 0.1–0.3 suggests that the odds of linkage to care among deferred high-risk prospective donors are between 70% and 90% lower than the odds of linkage to care among HIV-positive blood donors.

**Table 2:** Associations between donor characteristics and linkage to HIV treatment in Maputo City and Maputo Province, Mozambique

Covariate			Crude OR	Uncertainty	Adjusted OR	Uncertainty
Age group	13–19		—			
	20–29		1.3	(0.8–2.1)		
	30–39		1.0	(0.7–1.4)		
	40–64		1.0	(0.8–1.3)		
Sex	Female (baseline)		—			
	Male		0.7	(0.5–0.9)		
Donation venue	Permanent (baseline)		—			
	Mobile		1.1	(0.7–1.6)		
Service organization	SO1 (baseline)		—		—	
	SO2		0.4	(0.3–0.5)	0.4	(0.3–0.5)
	SO3		0.2	(0.1–0.3)	0.2	(0.1–0.3)
Donor	Positive (baseline)		—		—	
	Deferred		0.2	(0.1–0.3)	0.2	(0.1–0.3)

Among prospective blood donors who reported high-risk behaviors, young females were more likely to be lost to follow-up compared to young males (not shown). Donors and those deferred with a history of being treated for an STI and positive HIV test result had a 2.8 [0.93–12.32] and 2.4 [1.08–6.74] odds ratio respectively of being lost to follow-up at testing (Table 3).

**Table 3:** Crude odds ratios for potential risk factors for lost to follow up at testing site among prospective blood donors and deferred donors in Maputo City and Maputo Province, Mozambique

Risk factors for deferral	*N*	OR	95% uncertainty limits
HIV*-positive donor vs. deferred donor		0.97	(0.68–1.37)
Female vs. male gender		1.07	(0.79–1.46)
Age: 20–29 vs. 13–19		1.00	(0.60–1.62)
30–39 vs. 13–19		1.21	(0.82–1.82)
40–64 vs. 13–19		0.96	(0.73–1.26)
Sex with HIV positive partner	49	0.67	(0.37–1.21)
Sex with CSW*	56	1.00	(0.58–1.80)
Have tattoo(s)	12	1.12	(0.35–4.21)
Treated for STI(s)* in past 12 months	18	2.84	(0.93–12.32)
Prior positive HIV test result	26	2.49	(1.08–6.74)

*HIV  Human Immunodeficiency Virus, * CSW * Commercial Sex Worker, * STI * Sexually Transmitted Infections

The adjusted odds ratios in the final model (Table 3) indicate that CBOs SO2 and SO3 were not as successful as SO1 in linking HIV-positive donors to ART treatment. The true odds ratio for linkage by SO2 relative to SO1 is between 0.3 and 0.5 with 0.95 probability. The true odds ratio for linkage to care by SO3 relative to SO1 is between 0.1 and 0.3 with 0.95 probability. The odds of linkage to care among high-risk prospective donors who were deferred from donation was, with 0.95 probability, 0.1–0.3 times the odds of linkage among donors who contributed HIV-positive blood.

The probability of linkage of HIV-positive blood donors to treatment at SO 1 was less than 0.5, and the probability of linkage of deferred donors was 0.05–0.17 with 0.95 probability (Table 4). The probabilities of linkage of deferred donors were 0.02–0.08 from SO 2 and 0.01–0.04 from SO 3 with 0.95 probability.

**Table 4:** Probabilities for linkage of HIV-positive blood donors and prospective donors in Maputo City and Maputo Province, Mozambique

Service organization	Donor group	Linkage probability	95% Uncertainty limits	
SO1	Positive	0.42	(0.36–0.48)	
	Deferred	0.10	(0.05–0.17)	
SO2	Positive	0.23	(0.18–0.28)	
	Deferred	0.05	(0.02–0.08)	
SO3	Positive	0.12	(0.07–0.17)	
	Deferred	0.02	(0.01–0.04)	

## Discussion

We demonstrated the feasibility of using community-based organizations to improve blood donor notification of HIV status and linkage to HTS and HCT services at two of the largest blood donation sites in Mozambique.

Despite pre-donation screening being a long-standing practice, the implementation of the subsequent notification and linkage to care is difficult^24–26^. We had a 20% deferral rate of prospective blood donors, which falls within the range reported in similar studies: 22% (Jamaica), 11.3% (Ghana), 10% (India), and 6.3% (Pakistan)^19,27–29^. The use of behavioral screening pre-donation is a long-standing practice recommended by multiple blood transfusion experts^24–26^. However, implementing such behavioral screening questionnaires is difficult given the competing priority of NBTS, which does not wish to turn away potential donors. A total of 712 participants tested HIV positive despite being screened using the high-risk behavior questionnaire, raising concerns as to the effectiveness of the process to screen out seropositive donors. Furthermore, the efficacy of behavioral screening within the context of blood transfusion services is dependent on the algorithms used, the skill of the person screening, and strict adherence to the screening process^29,30^. The prospective blood donor’s responses can also affect the effectiveness of the behavioral screening, as some donors may know their HIV-positive status or other TTIs and choose not to disclose^10,20^. The site and manner in which the behavioral screening is conducted may influence its effectiveness, as demonstrated by a study conducted at a provincial hospital in Mozambique, which had a deferral rate of 9% versus 20% seen in our study, indicating the high variability of screening algorithms even within the same country^18^. Such variability could be explained by the lack of close supervision at field sites in comparison with that available at fixed sites, the availability of fewer staff at mobile sites, and time constraints. These findings suggest that mobile blood donation sites might benefit from implementing more stringent behavior screening protocols.

Literature shows that in several regions of the world, due to traditional, religious, and cultural reasons, men make up most of the blood donor pool^31–35^. The gender ratio of males (488) to females (224) in the study was approximately 2:1 and is noteworthy when considering strategies to identify, engage, and link men to HIV programs^36,37^. Additionally, as countries strive to reach the end of HIV as a public health threat by 2030, there are considerable efforts underway to engage men with HIV programming and attempts to identify points of contact with the healthcare system^36,38^. In most PEPFAR-supported countries, this gender imbalance among blood donors is similar and, as such, opens the possibility of transfusion centers and blood banks serving as an entry point for linking men to HIV and other health services^6,39^.

Blood donor notification is an essential component of an NBTS^40^. In high-resource settings where the technological systems are more advanced and low prevalence of blood-borne infections exist, the issue of donor notification plays a more minor role amidst the overall services provided^10,40^. In most high-resource settings, blood donors are informed of their test results via mail or telephone, a brief interview is conducted by the blood service, and they are referred to their primary care provider for the initiation of appropriate treatment. In such settings, donors are easily linked to further diagnosis, monitoring, and (when required) therapy^41–44^. The context and constraints to implementing such mechanisms are very different in low resource settings, particularly in Mozambique where the prevalence of HIV and other TTIs (HBV, HCV, syphilis) among blood donors ranged from 4.0% to 6.9% respectively^6^. The health system in Mozambique lacks the resources to follow up on donors and link them to services. Additionally, there are many challenges associated with tracing and tracking potential donors whose blood donation tested or screened HIV positive, such as lack of individual mobile cell phones, inefficient postal systems, mobile populations, shared housing, cost associated with mobile communication, and lack of precise addresses^10,12,40^. Thus, the existing donor notification system places the onus on the donors’ desires to know their infection status or disclose their HIV status. Additionally, the system does not offer HIV testing on site for those prospective donors who are screened out and deferred due to high risk. The role that HIV stigma and discrimination play in client disclosure and subsequent notification is seen with the increased odds of being lost to follow-up based upon risk behaviors (sex with commercial sex worker)^14,45,46^. The need for continued investments to eliminate the stigma associated with HIV disclosure remains a priority.

Several blood donor studies emphasized the importance of lack of clear communication during initial client visits and health communication messages as a factor in poor follow-up and donor notification^16,20,47^. However, despite the challenges mentioned above, the strategies used during this study demonstrated a higher notification rate (35%) compared to reports from similar studies conducted in Tanzania (less than 10%) and Burkina Faso (15%)^10,16^Models involving local community-based organizations working with the NBTS could be effective interventions for improving the linkage of deferred or positive donors to HCT services.

Our findings demonstrated higher linkage rates (20%) of HIV seropositive donors to ART initiation compared to a report from Ghana that saw only a 6% linkage to services^10,48^. The service organizations identified to link donors to HIV testing, care, and treatment services emphasized the difficulty associated with tracing, contacting, and connecting clients. The difficulties described by the service organizations associated with tracing blood donors, such as lack of resources, inadequate donor contact information, and long travel distances, were like those seen in a study conducted in Tanzania^10^. While the randomized assignment of participants to SBOs was meant to reduce biases, the varied success seen between the three service organizations in linking HIV-positive donors to ART treatment could be explained by factors such as the level of training of the community health workers and familiarity with communities within which they worked. Furthermore, the different methodologies used by the CSOs in linking clients and their differing outcomes highlight how a one-size-fits-all model may not be the most effective. Our findings emphasize the need for innovative approaches and methods by the NBTS, community-based organizations, and health systems to ensure notification of results and linkage to HCT of blood donors. Few studies have explored the use of community-based organizations to improve the linkage of HIV-positive donors and deferred donors to care and treatment^19,40^. Some SSA countries, such as Ghana and Gambia, identified similar barriers to donor notification and linkage to care and treatment of blood donor notification for other blood-borne pathogens^12,19^. Linkage to care plays a key role in the HIV care continuum and is an essential first step towards antiretroviral therapy initiation and viral suppression. Identifying successful strategies for notification and linkage services would impact cost savings and contribute towards the HIV treatment cascade.

## Limitations

Our study has several limitations. Firstly, our study sample is not representative of the entire country or of the rural population. Our study participants were mainly from the city of Maputo and its urban surroundings. Secondly, the majority of the donors in the study were males, which has implications as health-seeking behaviors vary between males and females. Third, the study was not designed to capture the seropositive status of all deferred participants. Finally, although the study implementation was adapted to leverage the use of existing community-based organizations involved in HIV linkage to care, this model is resource intensive and may not apply to all settings.

## Conclusion

In conclusion, the NBTS can facilitate linkage and follow-up of HIV-positive individuals and effectively refer high-risk individuals who are screened out of donation to prevention and testing services. Such an intervention deserves to be assessed at a larger scale in other low-resource settings where well-established NBTS exist and HIV prevalence remains a public health concern.

## Data Availability

The data sets generated during the study are available from the corresponding author on reasonable request.Data is provided within the manuscript.
